# Combination of everolimus and low-dose tacrolimus controls histological liver allograft injury as sufficiently as high-dose tacrolimus

**DOI:** 10.3389/frtra.2023.1168163

**Published:** 2023-04-20

**Authors:** Emily A. Bosselmann, Fabian Dranicki, Alejandro Campos-Murguia, Björn Hartleben, Heiner Wedemeyer, Elmar Jaeckel, Richard Taubert

**Affiliations:** ^1^ Department of Gastroenterology, Hepatology, Infectious Diseases and Endocrinology, Hannover Medical School, Hannover, Germany; ^2^ Institute for Pathology, Hannover Medical School, Hannover, Germany

**Keywords:** graft injury, fibrosis, mTOR inhibitor, everolimus, immunosuppression, tacrolimus

## Abstract

**Introduction:**

The combination of everolimus (EVR) and low-dose tacrolimus (lowTAC) prevents T cell-mediated rejection of liver grafts as sufficiently as high-dose tacrolimus (highTAC) and mycophenolate, but is associated with a preserved kidney function within the first years after orthotopic liver transplantation (OLT). However, none of the available studies assessed the histological pattern of graft injury or fibrosis in surveillance biopsies (svLbx).

**Methods:**

All svLbx taken under at least one month of stable immunosuppression with either EVR (aim 3-8 ng/ml) combined with lowTAC (aim 3-5 ng/ml) or highTAC (aim 5-8 ng/ml) combined with mycophenolate (500-1500 mg/day) within the first three to four years after OLT at our center were included. Patients who were switched to EVR because of insufficient control of alloreactivity were excluded.

**Results:**

Reasons for switches to EVR were mainly malignancies before or after OLT, or chronic kidney injury. We were able to include 20 svLbx with EVR/lowTAC and 49 with highTAC/mycophenolate. Both groups had similar liver enzymes and similar kidney function. The EVR/lowTAC group exhibited lower TAC trough levels at svLbx (4.4 vs. 6.6 ng/ml; p<.001) in comparison to highTAC/mycophenolate. Histological graft injury quantified by the rejection activity index and hepatitis activity index (Ishak), as well as fibrosis were not significantly different between the EVR/lowTAC and highTAC/mycophenolate groups. Likewise, subclinical TCMR, histological criteria justifying immunosuppression minimization, and steatosis had equal prevalence in both regimens. Immunosuppression was adjusted according to the svLbx findings. Immunosuppression regimens had similarly low rates of rejection after immunosuppression reduction, when relevant graft injury was absent in the biopsy.

**Discussion:**

In conclusion, EVR/lowTAC seems to control alloreactivity and histological graft injury as sufficiently as highTAC/mycophenolate within the first 3-4 years after OLT.

## Introduction

For some years now, mammalian target of rapamycin (mTOR) inhibitors such as everolimus (EVR) and sirolimus have been used in immunosuppressive therapy after orthotopic liver transplantation (OLT) (1). Regimens using a combination of low-dose tacrolimus (lowTAC) with an mTOR inhibitor have proven to be just as safe as high-dose tacrolimus (highTAC) combined with mycophenolate during the first year after OLT, at least when considering clinically overt graft rejection (2). However, we still lack data on the influence on subclinical graft rejection, meaning histological signs of rejection in patients with normal to near-normal liver enzymes. Previous studies have indicated that subclinical graft injury might be relevant for the progression of graft fibrosis (3). Also, Feng et al. were able to show that immunological tolerance and the possibility of immunosuppression (IS) minimization are influenced by subclinical graft inflammation (4, 5).

The gold standard in immunosuppression during the first year after OLT consists of steroids—which are usually discontinued after three to four months post-OLT—tacrolimus, and mycophenolate (6). Tacrolimus is dosed to reach trough levels of >5 ng/ml, while the standard dosage of mycophenolate is 1,000–1,500 mg/day.

As inhibitors of the mammalian target of rapamycin, which impede proliferation of T and B cells, two substances are available. Sirolimus has been used in immunosuppression after solid organ transplantation since 1999, when it was approved by the US Food and Drug Administration (FDA) (7). Everolimus has only been used for this purpose since 2012 (8). mTOR inhibitors work synergistically to calcineurin inhibitors (CNI) such as tacrolimus and cyclosporine A (9). Previous studies have shown that while mTOR monotherapy early after liver transplantation was associated with an increased rejection risk, the combination of mTOR inhibitors with low-dose CNI lead to less nephrotoxicity due to the fact that CNI dosages can be reduced compared to CNI monotherapy or combinations using mycophenolate instead of mTOR inhibitors (10–12). However, as far as we are aware, none of the prospective pharmacological studies included a systematic assessment of subclinical graft injury *via* svLbx, or at least liver stiffness measurements (11, 13–16).

Additionally, mTOR inhibitors can be used in patients transplanted for hepatocellular carcinoma, since they have been shown to have antiproliferative effects (17–21). A third indication for mTOR inhibitors is the reactivation of cytomegalovirus due to their antiviral effects (22).

For this study, svLbx performed in the context of the personalized IS program at our center were used. Histopathological findings were discussed in monthly interdisciplinary conferences and IS was then adjusted based on biopsy results and individual patient history (23).

This study aimed at comparing the occurrence of subclinical graft injury in surveillance liver graft biopsies (svLbx) in patients on high-dose tacrolimus (defined by a trough level aim of 5–8 ng/ml) and mycophenolate and patients on the regimen of low-dose tacrolimus (defined by a trough level aim of 3–5 ng/ml) with the mTOR inhibitor everolimus.

## Material and methods

### Subjects

We included all adult liver recipients without a replicative viral hepatitis (HCV-RNA or HBs-Ag negativity) who underwent a liver graft biopsy and agreed to participate in our prospective liver allograft biorepository database and in this prospective observational study from November 2018 to September 2022 (with ages at time of liver biopsy ≥18 years), as described recently (23, 24). Participation in the protocol biopsy program was voluntary and offered to all liver transplanted patients without contraindications, e.g., severely dilated bile ducts or thrombocytopenia. Only surveillance biopsies in patients with normal to near-normal liver enzymes [≤2× upper limit of normal (ULN)] were included in this analysis. Also, for this study, we only included biopsies performed within the first 45 months after OLT. To improve comparability, patients on immunosuppression regimens with cyclosporine A, for example because of tacrolimus intolerance or because of OLT for primary biliary cirrhosis, were excluded from the study, as well as patients on combinations of low-dose tacrolimus and mycophenolate (25, 26). Patients on regimens using EVR were excluded if EVR had been introduced due to insufficient control of alloreactivity under highTAC/mycophenolate (*n* = 3).

The study was approved by the Ethics Committee of Hannover Medical School, Hannover/Germany (protocol number 933 for project Z2 of comprehensive research center 738). Written informed consent was obtained from all subjects. All experiments were performed in accordance with relevant guidelines and regulations. No organs or tissues were procured from prisoners.

### Liver biopsy specimens

Liver biopsies were performed percutaneously and ultrasound-guided under local anesthesia with a 16-gauge needle, fixed in 4% neutral buffered formalin and embedded in paraffin wax, as described recently (23).

### Histological grading and staging

Histological grading and staging was performed as described recently (27). Sections of 2 µm thickness from liver allograft biopsies were stained with hematoxylin and eosin, elastic van Gieson stain, periodic acid–Schiff stain, silver stain, Berlin blue stain, and rhodanine stain. Histological examination was performed by experienced liver pathologists in a blinded fashion. Only Lbx regarded as representative by the examining pathologist, including at least five portal fields, were included. The liver tissue was examined regarding the Ishak scoring system, as well as—where possible—liver allograft fibrosis (LAF) score and Banff schema for grading liver allograft rejection with the rejection activity index (RAI). For the Ishak (mHAI) score, five different categories were examined: periportal or periseptal interface hepatitis (piecemeal necrosis; Ishak A; 0–4 points), confluent necrosis (Ishak B; 0–6 points), focal (spotty) lytic necrosis, apoptosis, and focal inflammation (Ishak C (0–4 points), portal inflammation (Ishak D; 0–4 points), and fibrosis stage (Ishak F; 0–6 points). The RAI score was constituted by examining portal, bile duct, and venous-endothelial inflammation with a maximum of three points, respectively (28). Patients with at least one point in each of the three categories, therefore showing morphological signs of graft rejection, and non-elevated liver enzymes (AST and ALT ≤2× ULN) were diagnosed with subclinical T cell-mediated rejection (subTCMR), as described previously by our group (23, 29). LAF was scored by separately assessing portal, sinusoidal, and centrilobular areas, each with a maximum of three points, allowing the maximum score to lie at nine points for both RAI and LAF (30, 31).

Histological criteria for the minimization of immunosuppression (BanffMini) was defined according to latest Banff consensus document (27, 28). In short, BanffMini was regarded to be fulfilled if all of the following criteria were met: portal tract inflammation ≤ 1, interface hepatitis ≤ 1, central perivenulitis ≤ 1, lobular inflammation = 0, biliary inflammation = 0, endothelialitis = 0, portal microvasculitis = 0, and periportal fibrosis ≤ 3, as described recently (23).

### Statistical analysis

Statistical analysis was performed using SPSS 15.0. The Mann-Whitney *U* test was used to compare quantitative data between two groups. The *χ*^2^ test was used to prepare contingency tables with two groups. *p*-values below 0.05 (two-tailed) were considered significant in all analyses.

Propensity Score Matching was used to address bias and balance the variables that could influence the probability of treatment assignment or the outcomes, in this case, age at liver biopsy, since younger patients are more likely to develop T cell-mediated rejection, and time after liver transplantation, since rejection risk is greater during the first months after liver transplantation (32). The propensity score was estimated using logistic regression. The matching procedure was performed using the nearest available neighbor, without replacement in a 1:1 ratio and without caliper. Standardized mean difference (SMD) lower than 0.1 was considered sign of balance (33, 34). This part of the statistical analysis was done using R version 4.1.2 with the MatchIt package for the PSM analysis (35).

Figures were generated using GraphPad Prism 9.5.0 and Affinity Designer 1.10.6.

## Results

**Figure 1:**
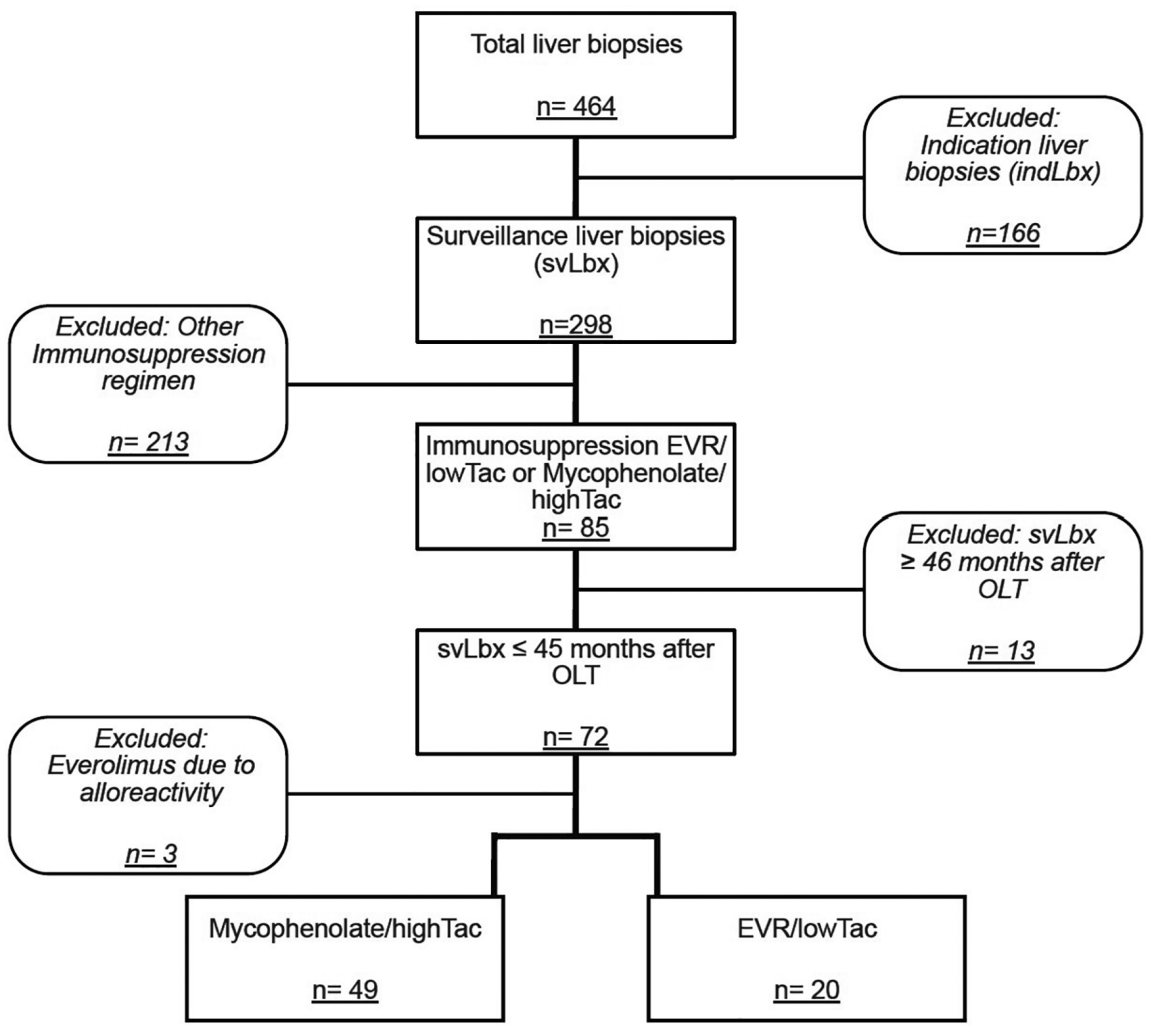
Flow chart outlining availability and selection of patients. The main selection criteria and grouping are outlined.

From November 2018 to September 2022, a total of 298 surveillance biopsies (svLbx), performed to assess subclinical graft injury in patients with normal to near-normal liver enzymes (≤2-fold ULN), were included in this study. No significant periinterventional complications were noted, as reported previously: no bleeding complications, no drops in hemoglobin levels, and no periprocedural infections (23). Of those patients, 69 had their first svLbx within the first 45 months after OLT while on a stable IS regimen of either highTAC/mycophenolate [*n* = 49 (71%)] or an alternative regimen with EVR/lowTAC [*n* = 20 (29%)] (Figure 1). The switch to EVR/lowTAC had been performed at a median of 6 (range: 1–25) months after OLT and 14 (range: 2–34) months before the liver biopsy. Reasons for switching the IS regimen to EVR/lowTAC included malignancies before or after OLT (*n* = 9; 45%), chronic kidney insufficiency (*n* = 6; 30%), intolerance of highTAC/mycophenolate (*n* = 3; 15%), and cytomegalovirus viraemia (*n* = 2; 10%). Baseline liver function tests, kidney function, and tacrolimus trough levels are shown in Table 1. Patients on EVR/lowTAC were shown to have been slightly older at OLT and at biopsy, while patients on highTAC/mycophenolate showed slightly higher bilirubin levels, mostly within the normal range. The two patient groups exhibited different tacrolimus trough levels (*p* < 0.001), while showing similar kidney function and liver enzymes at time of biopsy. Even though some patients were below their tacrolimus trough level target range (*n* = 5; 10%) in the highTAC cohort and some were above their target range (*n* = 7; 35%) in the EVR cohort at time of biopsy, we did not exclude these patients from the study. A detailed analysis showed that the majority of patients were within their tacrolimus target ranges for most of the time before svLbx (data not shown).

**Table 1:** Baseline characteristics of patients included in the study.

	EVR/lowTAC (*n* = 20)	highTAC/mycophenolate (*n* = 49)	*p*-values EVR/lowTAC vs. highTAC/mycophenolate
Age at biopsy (years)	56 (31–66)	51 (29–63)	**0**.**028**
Male gender, *n* (%)	11 (55)	32 (65)	0.423
BMI at biopsy (kg/m^2^)	23.5 (19.4–31.8)	24.5 (17.3–35.9)	0.455
Underlying disease, *n* (%)			0.233^1^
Autoimmune Liver Disease	4 (20)	14 (29)	
Chronic viral hepatitis	5 (25)	3 (6)	
Non-alcoholic fatty liver disease	3 (15)	2 (4)	
Alcoholic liver disease	5 (25)	6 (12)	
Cryptogenic	1 (5)	11 (22)	
Other	2 (10)	13 (27)	
Reason for OLT, *n* (%)			0.061^1^
Decompensated Cirrhosis	5 (25)	17 (35)	
Hepatocellular carcinoma	9 (45)	2 (4)	
Acute Liver Failure	1 (5)	6 (12)	
Acute-on-chronic Liver Failure	1 (5)	7 (14)	
Primary Sclerosing Cholangitis	3 (15)	5 (10)	
Other	1 (5)	12 (24)	
Age at OLT (years)	54 (30–63)	50 (26–61)	**0**.**022**
Time from OLT to biopsy (months)	18 (11–44)	18 (9–45)	0.895
AST (U/L)	25 (17–37)	22 (10–64)	0.427
ALT (U/L)	20 (11–43)	21 (7–84)	0.266
AP (U/L)	101 (55–308)	92 (32–412)	0.151
GGT (U/L)	33 (11–148)	28 (7–927)	0.534
Bilirubin (µmol/L)	6 (3–16)	9 (4–32)	**0**.**008**
Creatinine (µmol/L)	106 (63–229)	102 (64–246)	0.561
eGFR (ml/min/1.73)	56 (23–105)	63 (23–115)	0.138
Total cholesterol (mg/dl)	188 (131–298)	166 (104–232)	**0**.**003**
Tacrolimus trough level at biopsy (ng/ml)	4.4 (2.8–8.5)	6.6 (2.9–10.9)	**<0**.**001**
Everolimus trough level at biopsy (ng/ml)	4.85 (3.7–9.3)		** **
Mycophenolate dosage at biopsy (mg/day)		1,000 (500–1,500)	** **
Donor-specific anti-HLA antibodies, *n* (%)^2^	0	1 (4)	0.499

Values are described as median (range), unless indicated differently.

*p*-values <0.05 are printed in bold type.

*p*-values were calculated by Mann–Whitney *U*-test, except^1^.

^1^ *p*-values calculated by *χ*^2^ test.

^2^ Testing for DSA was performed in 12 patients on EVR/lowTAC and in 27 patients on highTAC/mycophenolate.

Patients on EVR/lowTAC had comparatively higher total cholesterol levels than patients on highTAC/mycophenolate (*p* = 0.003). Unfortunately, differentiation of cholesterol into HDL and LDL was available only in very few patients at baseline (EVR/lowTAC, *n* = 9 (45%): median HDL = 59 mg/dl, median LDL = 119 mg/dl; highTAC/mycophenolate, *n* = 11 (22%): median HDL = 52 mg/dl, medial LDL = 102 mg/dl). Baseline LDL was however significantly higher in the EVR/lowTAC group (*p* = 0.007). The difference in LDL levels lost significance (*p* = 0.086) at follow-up (FU), with a median of 123 mg/dl in the EVR/lowTAC cohort (*n* = 14/18, 78%) compared to 104 mg/dl in the highTAC/mycophenolate group (*n* = 26/45, 58%).

**Figure 2:**
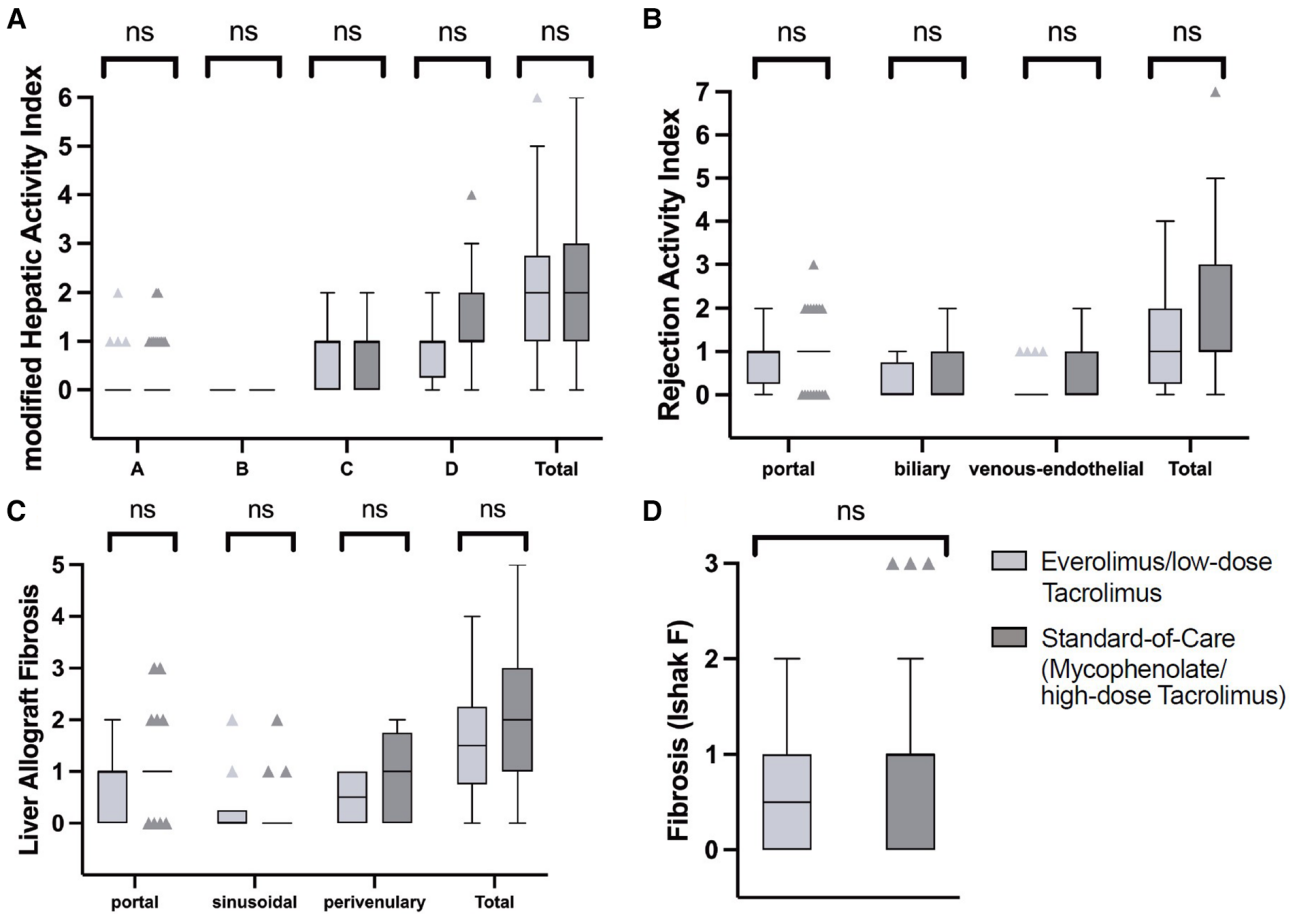
Histological findings. Histological findings were categorized and compared between the EVR/lowTAC (light grey) and mycophenolate/highTAC (dark grey) cohorts. (**A**) No significant (n.s.) differences between the groups were found using the modified Hepatic Activity Index (mHAI), indicating (subclinical) graft injury such as periportal or periseptal interface hepatitis (mHAI A), confluent necrosis (mHAI B), focal lytic necrosis, apoptosis, focal inflammation (mHAI C), and portal inflammation (mHAI D). (**B**) The Rejection Activity Index (RAI) was used to indicate (subclinical) graft rejection. No significant differences were found between the two cohorts. (**C**) The Liver Allograft Fibrosis (LAF) score and (**D**) the Ishak Fibrosis score were analyzed in both groups to categorize graft fibrosis. They also showed no significant differences between the cohorts.

Inflammation grade according to the modified Hepatic Activity Index (mHAI A–D) was also similar between the two groups, as well as the Rejection Activity Index (RAI), meaning portal inflammation (*p* = 0.246), biliary inflammation (*p* = 0.372), and venous-endothelial inflammation (*p* = 0.445) (Table 2, Figure 2). Likewise, the fibrosis scores were not significantly different between the two immunosuppression regimens. To exclude that the difference in patient age between the two groups (Table 1) biased the graft injury pattern, we added a propensity score matched analysis with a one-to-one ratio including the patient age (Supplementary Figure S1, Table S1). Graft injury patterns were still not significantly different when comparing these smaller but matched immunosuppression groups (Supplementary Tables S2, S3). Interestingly, there was a trend towards less periportal fibrosis (*p* = 0.067) and less portal inflammation (*p* = 0.054) in the EVR/lowTAC group (Supplementary Table S2).

**Table 2:** Histological findings—inflammation.

	EVR/lowTAC (*n* = 20)	highTAC/mycophenolate (*n* = 49)	*p*-values EVR/lowTAC vs. highTAC/mycophenolate
Ishak A	0 (0–2)	0 (0–2)	0.847
Ishak B	0	0	1.0
Ishak C	1 (0–2)	1 (0–2)	0.931
Ishak D	1 (0–2)	1 (0–4)	0.345
RAI portal	1 (0–2)	1 (0–3)	0.246
RAI biliary	0 (0–1)	0 (0–2)	0.372
RAI venous-endothelial	0 (0–1)	0 (0–2)	0.445

Values are described as median (range).

In the following, we refer again to the unmatched cohorts. Seven (35%) biopsies from patients on the EVR/lowTAC scheme and 11 (22%) from patients on highTAC/mycophenolate showed the absence of or only mild histological inflammation within the histological criteria justifying IS minimization (BanffMini) according to the 2016 Banff consensus document (*p* = 0.285) (28). Subclinical T cell-mediated rejection (subTCMR), defined as RAI of at least one point for each of the assessed areas (portal, biliary, and venous-endothelial), was detected in three (15%) patients on EVR/lowTAC regimen and in 10 (20%) patients on highTAC/mycophenolate, showing no significant difference between the groups (*p* = 0.605). Overall relevant graft fibrosis, defined as Ishak F ≥ 2, was detected in eight (12%) biopsies, twice in patients on EVR/lowTAC (10%) and in six patients on highTAC/mycophenolate (12%). The detection of relevant graft fibrosis was not significantly different between patients on either IS regimen (*p* = 0.351). The Liver Allograft Fibrosis (LAF) score, which was only available in 34/69 biopsies, did not show any significant differences between the groups, either (LAF portal: *p* = 0.360; LAF sinusoidal: *p* = 0.724; LAF centrilobular: *p* = 0.467).

Significant graft steatosis, defined as the accumulation of droplets of fat in >5% of hepatocytes, was detected in 14 (20%) biopsies. Three (15%) patients on EVR/lowTAC and 11 (22%) patients on highTAC/mycophenolate showed steatosis (*p* = 0.488). Histological findings are also presented in Table 3.

**Table 3:** Histological findings—diagnoses.

	EVR/lowTAC (*n* = 20)	highTAC/mycophenolate (*n* = 49)	Total cohort (*n* = 69)	*p*-values (EVR/lowTAC vs. highTAC/mycophenolate)
Fibrosis (Ishak F2-4), *n* (%)	2 (10)	6 (12)	8 (12)	0.351
Cirrhosis (Ishak F5-6), *n* (%)	0 (0)	0 (0)	0 (0)	
Subclinical T cell mediated rejection, *n* (%)	3 (15)	10 (20)	13 (19)	0.605
Graft Steatosis *n* (%)	3 (15)	11 (22)	14 (20)	0.488
BanffMini*, *n* (%)	7 (35)	11 (22)	18 (26)	0.285
Disease recurrence, *n* (%)	0	0	0	

Percentages are shown in brackets.

* Fulfillment of histological criteria justifying immunosuppression minimization according to Banff consensus (28).

Testing for donor-specific anti-HLA antibodies (DSA) within a time range of 12 months around svLbx was available in 39 (57%) patients. DSA frequency was not different between both treatment regimens (Table 1).

All biopsies included in this study were also included in our center's program on the individualization of immunosuppression in liver transplant recipients (23). A total of 18 (26%) biopsies fulfilled the histological criteria justifying immunosuppression minimization according to the 2016 Banff consensus document (BanffMini) (28). Even though many of them did not fulfill BanffMini criteria, the reduction of IS was advised in our interdisciplinary conference in 50 (72%) patients, of whom eight were in the EVR/lowTAC cohort and 42 were on highTAC/mycophenolate. Reduction of IS was successfully performed, meaning the changes were kept without signs of alloreactivity until the FU visit to our center at a median of 14 (range: 8–40) months after the biopsy, in four (80%) patients on EVR/lowTAC and 31 (86%) patients on highTAC/mycophenolate. These results were similar to our previous analysis of the first two to three years of the whole personalized immunosuppression program (23).

Nine patients (four on EVR/lowTAC and six on highTAC/mycophenolate) had not yet had their FU visit at our center until the finishing of the manuscript.

Three (7%) patients showed signs of rejection (liver enzymes >2× ULN) after IS reduction. Again, this frequency is similar to our previous analysis of the whole personalized immunosuppression program (23). All three patients were in the highTAC/mycophenolate group at time of biopsy. One (33%) of them had a svLbx result fulfilling BanffMini criteria, however, this patient had a biopsy-proven TCMR at around 6 months after IS reduction. The other two did not have a biopsy confirming alloreactivity. In all three cases, IS was increased again, and the patients showed (near-)normal (<2× ULN) liver enzymes at FU.

Other reasons for changes in the advised IS regimen were comorbidities in need of steroid therapy (ulcerative colitis, *n* = 1), non-compliance (*n* = 1), intolerance of therapy (*n* = 1), and other organ transplantation (*n* = 1).

In the EVR/lowTAC group, EVR was discontinued due to the biopsy results in three (15%) patients. Two of these patients (67%) were switched to tacrolimus monotherapy. The third changed to a lowTAC/mycophenolate scheme due to side effects of EVR (edema). In the highTAC/mycophenolate group, eight (16%) patients were switched to EVR after their biopsies. In three (38%) of them, the change to EVR was made due to significant graft fibrosis (Ishak F ≥ 2 and/or LAF score ≥ 3) according to our local practice (23). The other five (62%) were switched due to kidney insufficiency.

At time of biopsy, baseline kidney function was not significantly different in the highTAC/mycophenolate cohort than the EVR/lowTAC cohort (63 ml/min/1.73 vs. 56 ml/min/1.73, respectively; *p* = 0.171).

## Discussion

In the past, different studies have been able to show that EVR/lowTAC is just as safe as highTAC/mycophenolate in OLT recipients when it comes to biopsy-proven acute TCMR during the first year after OLT (1, 2, 13–15). Also, the mTOR inhibitors' efficacy has been proven, showing that a combination of low-dose tacrolimus and an mTOR inhibitor can improve renal function at early time points after OLT (10–12). A study from our own center proved that reduction of CNI trough levels can not only stabilize, but even improve kidney function significantly even beyond year one (23).

In this current study, we were able to show that in addition to the prevention of clinically overt TCMR in the abovementioned pharmacological studies, subclinical graft injuries including subclinical TCMR only detected through svLbx are also not increased in patients on EVR/lowTAC compared to highTAC/mycophenolate. Inflammation grades according to mHAI and RAI were similar in the two groups. In median, both treatment regimens exhibited patterns of mild graft injury. Also, the groups did not differ regarding the BanffMini criteria and graft fibrosis. Notably, even though patients on EVR/lowTAC showed significantly higher total cholesterol and LDL levels, matching previous studies on the subject, both groups showed similar amounts of graft steatosis (36–38). The age difference between the patient groups (56 years 321 in the EVR/lowTAC group vs. 51 years in the highTAC/mycophenolate group; *p* = 0.028, 322 Table 1) was a possible confounder variable to our results. We therefore performed an additional propensity score matched analysis in which the age difference did not reach significance and which found similar graft injury patterns in both matched groups.

As far as we are aware, ours is the first center to make this analysis using svLbx. Cholesterol levels in patients on any EVR regimen should however be closely monitored, since dyslipidemia, as a known side effect of mTOR inhibitors, increases the risk of cardiovascular diseases and complications, such as atherosclerosis and myocardial infarction (39). In order to reduce cardiovascular complications in patients receiving EVR, cholesterol reducing drugs, such as statins, may be started according to existing guidelines before transplantation (40).

We saw no difference in safety of IS withdrawal following svLbx between the groups. It is notable however, that biopsy-proven TCMR and significant elevation of liver enzymes were seen only after IS reduction in the highTAC/mycophenolate cohort, even though this did not reach significance between the groups. Interestingly, an intentional and complete IS withdrawal was more successful in OLT recipients on mTOR therapy than on other regimens in previous studies (41). However, further studies are needed to explore whether mTOR inhibitors are superior in inducing transplant tolerance.

Although many centers do not perform svLbx on a regular basis for various reasons (e.g., for fear of complications or for lack of capacities), in this current study, svLbx appeared to be safe, seeing as no relevant complications, such as bleeding, were noted. This matches previous data from our center, where svLbx have been performed since 2008 (23, 42). However, a growing number of studies demonstrate that at least moderate subclinical graft injuries, not mirrored by elevation of liver enzymes above the upper limit of normal and only detectable by svLbx, are relevant for the risk assessment. More advanced subclinical graft injuries, including subTCMR, are associated with a higher expression of rejection-associated transcripts in the liver graft, which is associated with fibrosis progression (3, 5, 24, 43). In addition, the presence of donor-specific anti-HLA antibodies is associated with a higher expression of rejection-associated transcripts, further underlining their role as non-invasive markers of graft injury (27, 44).

The histological findings of this study, with only few biopsies fulfilling the criteria justifying a minimization of IS defined in the 2016 Banff consensus document, also match previous findings from our own and other centers (3, 5, 23, 27, 45).

As described previously, our center attempts for a stepwise IS reduction following svLbx without relevant graft injury (23). This method was applied in this study, as well, showing again that svLbx can be useful in deciding on the adequate IS regimen for each individual patient. Although three patients had elevated liver enzymes after IS reduction, only one patient had biopsy-proven TCMR and liver values were normalized by re-increasing IS in all three patients. These frequencies are within the range of our previous analysis of our program (23). This shows that a svLbx-guided IS reduction is safe in terms of graft loss or steroid-resistant TCMR after EVR/lowTAC just as after highTAC/mycophenolate, as long as specific measures, such as regular outpatient visits and close monitoring of liver enzymes, are taken. Of course, these results must be interpreted with caution, since the follow-up period was still quite short, even though this matches previous IS withdrawal studies (46, 47).

Obvious limitations of the current study are that it is a single center retrospective evaluation of a clinical practice and not a randomized controlled study. Patient numbers are limited, especially in the EVR/lowTAC cohort. This may be partly due to the fact that EVR has only been approved for OLT recipients for a few years, for it is also a medication with frequently occurring adverse effects, meaning that at our center, up to a third of patients wish to discontinue EVR even after short time periods. Since EVR has only been approved for OLT in Germany for a few years, we still lack safety data regarding its long-term effects, data that in contrast is available for CNI. mTOR inhibitors are associated with more side effects with intolerance rates of 20%–30%. Therefore, at our center, we switch patients to EVR only for certain reasons, such as contraindications for high-dose tacrolimus (e.g., kidney insufficiency), intolerance of mycophenolate (e.g., leucopenia), HCC before OLT, or CMV reactivation. Since this is not a randomized controlled study and EVR is begun for explicit reasons at our center, that means we also have a selection bias between the cohorts towards older patients with a higher prevalence of HCC in the EVR/lowTAC group. Furthermore, the time point of switch to EVR in this real-life cohort is rather heterogenous. Although we have a high number of patients having undergone OLT for autoimmune liver diseases, these patients are rather left on highTAC/mycophenolate, compared with patients transplanted for malignant diseases, such as hepatocellular carcinoma, which gives us reason to discontinue highTAC/mycophenolate and switch to EVR/lowTAC due to the mTOR inhibitors' antiproliferative effects (17–21). However, the patient number was too low for a more precise matching of the cohorts. The fact that EVR has been approved for some years now also means that all necessary approval studies have already been performed, so financial support for a randomized, controlled study repeating the previous studies only for the purpose of looking at subclinical graft injury in svLbx is not very likely. So, retrospective analysis of prospectively collected real-life data will be the best available data source in the near future. However, multicenter confirmation of the present monocentric results are mandatory. Finally, a high intra-individual variability of dose-to-trough-level ratio, which is known in CNI, was observed in this real-life cohort study. Such a variability was even seen in randomized controlled trials. We therefore included all patients on the basis of their trough levels because patients were within their trough level aim in the majority of available measurements.

In summary, the present study complements previous pharmacological studies by showing a good safety profile of EVR/lowTAC even in terms of subclinical graft injury. Even though a further, multicenter analysis and longer follow-up periods with repeated svLbx appear reasonable, this study shows that EVR/lowTAC is safe at least in the first three to four years after OLT even in reference to all histopathological—and not only to clinically overt—findings. The combination of EVR/lowTAC was also shown to be a comparable baseline for further IS reduction without risking more acute rejection than after highTAC/mycophenolate.

## Data Availability

The raw data supporting the conclusions of this article will be made available by the authors, without undue reservation.
