# Supplementary figures and images for: Combination of everolimus and low-dose tacrolimus controls histological liver allograft injury as sufficiently as high-dose tacrolimus
Source: Front Transplant. 2023 Apr 20;2:1168163. doi: 10.3389/frtra.2023.1168163 (PMC11235273; doi:10.3389/frtra.2023.1168163)

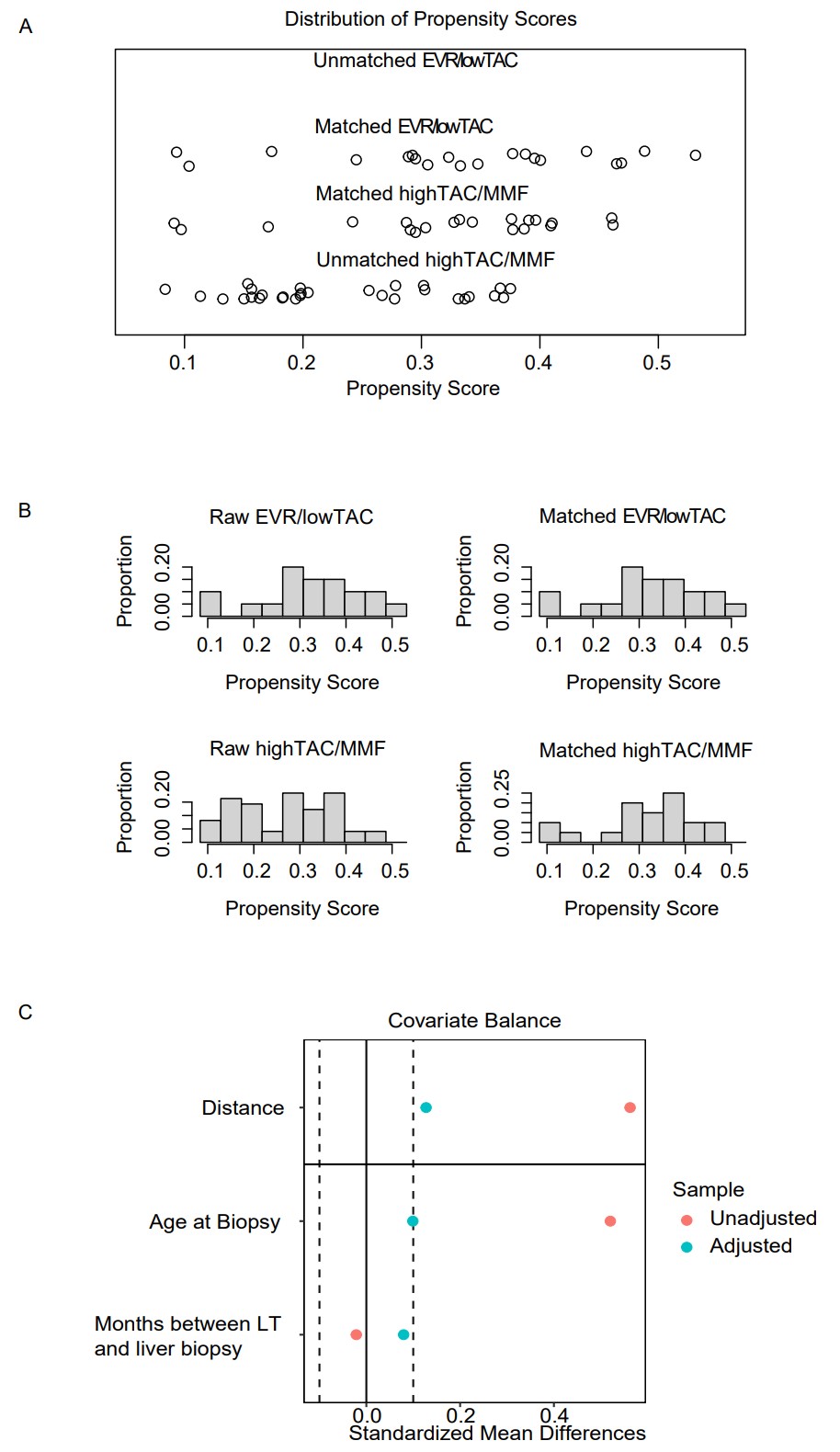

Supplement: Supplementary file 1 [file Image1.jpeg]
