# Supplementary material for: Combination of everolimus and low-dose tacrolimus controls histological liver allograft injury as sufficiently as high-dose tacrolimus
Source: Front Transplant. 2023 Apr 20;2:1168163. doi: 10.3389/frtra.2023.1168163 (PMC11235273; doi:10.3389/frtra.2023.1168163)
Supplement: Supplementary file 2 [file Datasheet1.docx]

**Supplementary Material**

**Supplementary Figures**

**Supplementary Figure 1. Data of the PSM, age at liver biopsy and time (months after liver transplantation) as covariates.** (A) Distribution of matched and unmatched subjects of each group relative to their propensity score. Treated Units are those patients in the Everolimus/LowTAC, and control Units are those patients in the HighTAC/Mycophenolate. (B) Proportion of the sample before and after the PSM relative to the propensity score. Raw Treated are those patients in the Everolimus/LowTAC, and Raw Control are those patients in the HighTAC/Mycophenolate. (C) Data of the covariate balance, each point represents the Standardized Mean Difference of the corresponding covariate before (Unadjusted) and after (Adjusted) the PSM. A Standardized Mean Difference < 0.1 represents a proper balance.

**Supplementary Tables**

Supplementary Table 1: Baseline characteristics of patients of the PSM cohort

|  | | EVR/lowTAC | highTAC/  mycophenolate | | p-values |
| --- | --- | --- | --- | --- | --- |
|  | | (n = 20) | (n = 20) | |  |
| Age at biopsy (years) | | 55 [31, 66] | 55 [29, 63] | | 0.755 |
| Male gender n (%) | | 11 (55) | 13 (65) | | 0.747 |
| BMI at biopsy (kg/m^2^) | | 23.5 [19.4, 31.8] | 25.2 [18.5, 35.3] | | 0.172 |
| Underlying disease n (%) | |  |  | | 0.309 |
|  | Autoimmune liver disease | 5 (25) | 2 (10) | |  |
|  | Chronic viral hepatitis | 4 (20) | 5 (25) | |  |
|  | Non-alcoholic fatty liver disease | 5 (25) | 2 (10) | |  |
|  | Alcoholic liver disease | 1 (5) | 4 (20) | |  |
|  | Cryptogenic | 3 (15) | 2 (10) | |  |
|  | Other | 2 (10) | 5 (25) | |  |
| Reason for OLT n (%) | |  |  | | 0.038 |
|  | Acute-on-chronic liver failure | 1 (5.0) | 4 (20.0) | |  |
|  | Acute liver failure | 1 (5.0) | 1 (5.0) | |  |
|  | Decompensated cirrhosis | 5 (25.0) | 11 (55.0) | |  |
|  | Hepatocellular carcinoma | 9 (45.0) | 1 (5.0) | |  |
|  | Other | 1 (5.0) | 2 (10.0) | |  |
|  | Primary sclerosing cholangitis | 3 (15.0) | 1 (5.0) | |  |
| Age at OLT (years) | | 54 [30, 63] | 52 [26, 61] | | 0.674 |
| Time from OLT to biopsy (months) | | 18 [11, 44] | 17 [9, 45] | | 0.882 |
| AST (U/l) | | 25 [17, 37] | 23 [10, 60] | | 0.579 |
| ALT (U/l) | | 20 [11, 43] | 21 [7, 65] | | 0.675 |
| AP (U/l) | | 101 [55, 308] | 118 [56, 216.00] | | 0.636 |
| GGT (U/l) | | 33 [11, 148] | 33 [8, 927] | | 0.776 |
| Bilirubin (µmol/l) | | 6 [3, 16] | 10 [4, 31] | | 0.067 |
| Creatinine (µmol/l) | | 106 [63, 229] | 107 [76, 246] | | 1.000 |
| eGFR (ml/min/1.73) | | 56 [23, 105] | 58 [23, 103] | | 0.598 |
| Total cholesterol (mg/dl) | | 188 [131, 298] | 170 [104, 232] | | 0.085 |
| Tacrolimus trough level at biopsy (ng/ml) | | 4.4 [2.8, 8.5] | 6 [2.9, 10.7] | | 0.004 |
| Everolimus trough level at biopsy (ng/ml) | | 4.9 [3.7, 9.0] | 0 | | <0.001 |
| Mycophenolate dosage at biopsy (mg/d) | | n.a. | 1000 [500,1000] | | **-** |
| Donor-specific anti-HLA antibodies n (%)^2^ | | 0 | 0 | - | |

Data is provided as No. (%) or median [range].

Supplementary Table 2: Histological findings - Inflammation in the PSM cohort

|  | EVR/lowTAC  (n = 20) | highTAC/  mycophenolate  (n = 20) | p- values |
| --- | --- | --- | --- |
| Ishak A | 0 [0, 2] | 0 [0, 2] | 0.453 |
| Ishak B | 0 [0, 0] | 0 [0, 0] | 1.000 |
| Ishak C | 1 [0, 2] | 1 [0, 2] | 0.759 |
| Ishak D | 1 [0, 2] | 1 [0, 4] | 0.275 |
| LAF Total | 2 [0, 4] | 1 [1, 5] | 0.377 |
| RAI portal | 1 [0, 2] | 1 [0, 3] | 0.054 |
| RAI biliary | 0 [0, 1] | 0 [0, 2] | 0.789 |
| RAI venous-endothelial | 0 [0, 1] | 0 [0, 2] | 0.938 |

Data is provided as median [range].

Supplementary Table 3: Histological diagnoses in the PSM cohort

|  | EVR/lowTAC  (n = 20) | highTAC/  mycophenolate (n = 20) | p- values |
| --- | --- | --- | --- |
| Fibrosis (Ishak F2-4) n (%) | 2 (10) | 3 (15) | 1.000 |
| Cirrhosis (Ishak F5-6) n (%) | 0 | 0 | 1.000 |
| Subclinical T cell mediated rejection n (%) | 3 (15) | 3 (15) | 1.000 |
| Graft Steatosis n (%) | 3 (15) | 8 (40) | 0.157 |
| BanffMini n (%) | 7 (35) | 6 (30) | 1.000 |
| Disease recurrence n (%) | 0 | 0 | 1.000 |

Data is provided as No. (%)
